# Bioactive Hydrogel Based on Collagen and Hyaluronic Acid Enriched with Freeze-Dried Sheep Placenta for Wound Healing Support

**DOI:** 10.3390/ijms25031687

**Published:** 2024-01-30

**Authors:** Julia Sadlik, Edyta Kosińska, Dagmara Słota, Karina Niziołek, Agnieszka Tomala, Marcin Włodarczyk, Paweł Piątek, Jakub Skibiński, Josef Jampilek, Agnieszka Sobczak-Kupiec

**Affiliations:** 1 Department of Materials Science, Faculty of Materials Engineering and Physics, Cracow University of Technology, 37 Jana Pawła II Av, 31-864 Krakow, Poland; 2 Department of Immunology and Infectious Biology, Faculty of Biology and Environmental Protection, University of Lodz, Banacha 12-16, 90-237 Łódź, Poland; 3 Department of Immunogenetics, Medical University of Lodz, ul. Pomorska 251/A4, 92-213 Łódź, Poland; 4 BioMedChem Doctoral School of University of Lodz and Institutes of the Polish Academy of Sciences, University of Lodz, Matejki 21/23, 90-237 Łódź, Poland; 5 Department of Analytical Chemistry, Faculty of Natural Sciences, Comenius University, Ilkovicova 6, 842 15 Bratislava, Slovakia; 6 Department of Chemical Biology, Faculty of Science, Palacky University Olomouc, Slechtitelu 27, 783 71 Olomouc, Czech Republic

**Keywords:** hydrogel, hyaluronic acid, placenta, collagen, composites, wound healing

## Abstract

In an increasingly aging society, there is a growing demand for the development of technology related to tissue regeneration. It involves the development of the appropriate biomaterials whose properties will allow the desired biological response to be obtained. Bioactivity is strongly affected by the proper selection of active ingredients. The aim of this study was to produce bioactive hydrogel materials based on hyaluronic acid and collagen modified by the addition of placenta. These materials were intended for use as dressings, and their physicochemical properties were investigated under simulated biological environmental conditions. The materials were incubated in vitro in different fluids simulating the environment of the human body (e.g., simulated body fluid) and then stored at a temperature close to body temperature. Using an FT-IR spectrophotometer, the functional groups present in the composites were identified. The materials with the added placenta showed an increase in the swelling factor of more than 300%. The results obtained confirmed the potential of using this material as an absorbent dressing. This was indicated by pH and conductometric measurements, sorption, degradation, and surface analysis under an optical microscope. The results of the in vitro biological evaluation confirmed the cytosafety of the tested biomaterials. The tested composites activate monocytes, which may indicate their beneficial properties in the first phases of wound healing. The material proved to be nontoxic and has potential for medical use.

## 1. Introduction

Despite significant advancements in medicine, the number of people suffering from epidermal damage caused by burns, ulcers, and other traumatic accidents leading to chronic wounds and infections is increasing every year [1]. The issues of our times have led us to seek new solutions, including materials that can replace and heal organs and tissues. 

The skin is regarded as the largest organ of the human body. It is important for us to protect ourselves from outside forces [2]. The skin is a highly sensitive tissue, as it is the organ that is most exposed to the external environment. Although many skin injuries can be easily treated to restore the original appearance of the tissue, certain injuries take longer to heal and result in the formation of scar tissue [3,4]. Damage to the integrity of the skin may result from various factors. Some wounds remain hard to heal, especially for people with cancer, autoimmune conditions, the elderly, and bedridden individuals at risk of bedsores or burns [5,6]. Wounds are often associated with inflammation, which is an increasingly common problem in today’s world. Regardless of the type of inflammation, it is defined as the body’s physiological response when trying to get rid of harmful agents and restore tissue homeostasis. This can result in irreversible damage to infected tissues [7,8]. Therefore, the introduction of innovative new dressings that promote the healing of even chronic wounds while reducing healthcare costs is an important issue [9].

In the presented situations, high-quality hydrogel biomaterials come to the rescue, relieving the patient’s pain [10]. In recent years, scientists have been working on developing bioengineered materials for wound healing purposes. For instance, they have been working on creating chitosan/polyvinyl alcohol (PVA) honey hydrogel films that could be used for treating wounds. Moreover, researchers have also developed lignin nanoparticle (LNP) constructs that have potential applications in the field of medicine [1,9]. Materials of this type contribute to keeping the wound moist, thus speeding up the healing process, and are fully flexible, which allows them to be applied to hard-to-reach areas [6]. 

In this case, the desired feature is bioactivity, which would accelerate tissue regeneration and shorten the patient’s recovery. Bioactivity can be ensured by selecting the right composition of such composites, including active ingredients, growth factors, or antibiotics that stimulate the tissue [5]. When creating a material for wounds, i.e., for tissue disruption and damage to the epidermis and deeper tissues, scientists are guided by the selection of the appropriate materials that not only allow proper tissue fusion to prevent scarring but also relieve pain and give comfort to the patient [11,12]. It is important to note that monocyte activity plays a crucial role in the initial stage of wound healing. Hence, it is necessary to assess the activation of the NF-κB factor in monocytes when developing new dressings. This helps determine the biomaterials’ ability to stimulate immune cell activation [5]. Nowadays, hydrogel materials are in high demand. They are widely used in branches of medicine as drug carriers, cell scaffolds, or surgical fillers. The field of dressings and transdermal systems is developing extensively. There are several criteria that such a dressing must meet in order to be considered:

- Creates an optimal environment (thermoregulation, suitable humidity, gas exchange, and pH);
- Facilitates the removal of exudate;
- Is nontoxic;
- Does not adhere to the traumatic wound;
- Is transparent and controls the healing process [13].

A hydrogel is a substance whose gelling agent is a polymer, which can be either synthetic or natural, and whose dispersed phase is water, which creates three-dimensional networks. These materials have the ability to absorb large amounts of water; moreover, this can be a reversible process. The structure of hydrogels gives them the characteristics of both liquids and solids. Hydrogel materials can be divided according to various parameters—the origin of the polymer used to build the network, the type of interaction between polymer chains, or the type of substance used to build the network [14].

The basic property described for this type of material is the ability to absorb water or biological fluids. The next important parameter is the determination of functional groups, i.e., whether they are hydrophilic or hydrophobic. The structure of the hydrogel depends on the method of its preparation. There are many ways to produce hydrogels; the main division is between chemical and physical [15,16,17].

Hyaluronic acid (HA) is a natural polymer belonging to a group of heteropolysaccharides named glycosaminoglycans [18]. It can bind large amounts of water due to its anionic structure, which has an affinity for cations, and this amounts to about 250 water molecules per molecule of HA [19]. It can be found in the human body, including in cartilage and joint lubricant, ensuring that joints are properly lubricated. The compound can also be found in the eye, specifically the vitreous body, as well as in the brain. The presence of this acid is important for the vocal cords and in otolaryngology in general. Topical preparations accelerate the healing of fresh wounds, as well as burns and diabetic wounds. It has a significant role due to its anti-inflammatory properties, stimulating cellular migration and ensuring wound hydration [19,20,21,22].

Collagen (COL) makes up as much as 30% of human protein weight, making it one of the most important proteins. It is found in many parts of the human body, including the skin, tendons, and bone, as well as cartilage. Collagen is made of amino acids such as glycine, proline, hydroxylysine, and hydroxyproline, and its structure varies depending on its occurrence and role [23]. The most important function of COL is to provide structural support, and cell-cell interactions. Other very important roles of COL include its participation in system cell adherence and its ability to bindtoxins and microorganisms, thus preventing their spread. Thanks to its ability to bind water, it keeps the skin hydrated, contributing to the regeneration of connective tissue and thus wound healing. COL is also known for its role as a drug carrier for interferon [24,25,26]. 

The placenta is an organ found in female mammals that enables the extension of the species. Its primary function is to nourish the embryo with oxygen and nutrients and remove metabolic products [27]. It also contains immune cells, antibodies, cytokines, growth factors, and glycoproteins. Therefore, the statement that it is a natural treasure is commonly circulated. We can find information about the use of the placenta by delving into the Asian culture, where the consumption of the placenta was a common thing. It was not just the Chinese culture that was enthralled with its valuable properties; the practice of placenta consumption also reached North America in the 1970s [28]. It should also be noted that the placenta, which is typically discarded as biological waste, is of interest in the tissue bank, especially because of the amniotic membrane. It is rich in nutrients and not highly immunogenic, which is often used as a substitute for the healing of the skin and eye surface [29]. Until now, such use has been effective and popular in patients with shallow wounds resulting from burns and toxic or mechanical injuries; amniotic transplantation using the placenta is one of the primary surgical interventions [21,22]. Another important feature in the context of the valuable properties of the placenta with an application to wounds is its antioxidant properties [30,31]. Experimental studies in animal models have proven that it reduces signs of fatigue and increases resistance to stress [32].

## 2. Results

### 2.1. Electrochemical Analysis

#### 2.1.1. Potentiometry Analysis

**Figure 1:**
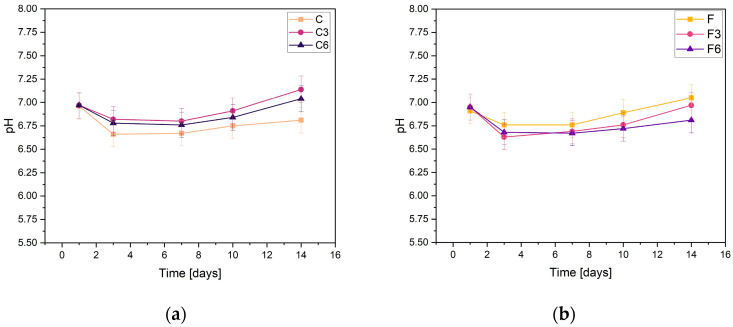
Measured pH values of composites containing (**a**) 0.15 g or (**b**) 0.30 g of placenta incubated in PBS solution.

During incubation, pH changes were studied to determine the stability of the fabricated biomaterials under conditions similar to those of the human body. The results are shown in Figure 1. They indicate slight variations in pH values. All samples behave in a relatively similar manner, regardless of COL and placenta content. The results presented confirm the buffering nature of the medium and the maintenance of the pH at a practically constant level. Slight fluctuations in pH measurements may result from temperature differences because the samples were incubated at 36.6 °C, whereas the measurement was made at a laboratory station where the temperature was 25 °C.

From the results obtained from the samples containing 0.15 g of placenta, it can be concluded that, in their case, the addition of COL, regardless of the amount, does not affect the changes in the pH values of the solution. After the first day of incubation, the pH slightly decreased and then stabilized. The pH values of all samples only slightly changed, fluctuating between 6.67 and 7.14. The highest pH values in every sample were observed after the 14th day of incubation in PBS solution. The sample C3 with a COL content of 0.3 g had the highest value on this day, while the composite without added COL had the lowest value. The slight pH fluctuations in PBS are probably due to the buffer capacity of the physiological fluid.

For the samples with increased placental content, the pH values were remarkably similar regardless of the amount of COL biomaterials. After the initial day of incubation, there was a minor decline in pH levels, which then achieved a state of stability. The highest pH value of 7.05 was recorded for sample F, which did not have COL added. In contrast, the composite with the addition of 0.03 g COL had the lowest pH value.

From the results, it can be concluded that the biomaterials produced are stable in PBS. This is supported by the absence of notable pH changes during the incubation process. Analogous pH relationships that indicate the stability of hydrogel materials were reported by Slota et al. [33]. Similar findings were observed for the samples both with and without COL; the curves of these materials were almost indistinguishable. However, minor pH fluctuations were present in group F samples, which contained a greater amount of placenta. Overall, all of the biomaterials tested exhibited comparable behaviors in PBS solution.

#### 2.1.2. Conductivity Analysis

**Figure 2:**
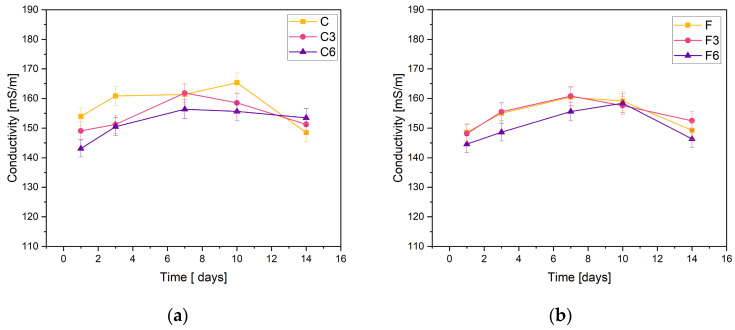
Measured conductivity values of composites containing (**a**) 0.15 g or (**b**) 0.30 g of placenta incubated in PSB solution.

The obtained materials were subjected to electrolytic conductivity tests after the incubation process. Figure 2 demonstrates the obtained results. The basis of the test is the change in ion concentration in the PBS solution.

The stability of the synthesized composites was confirmed through the measurements of C samples containing 0.15 g of placental content. The conductivity measurements recorded for the samples did not fall below 140 mS/m or exceed 170 mS/m. The sample without added COL showed an increase in conductivity on day 10 of incubation, while the other materials peaked on day 7. On the last day of incubation, a slight decrease in conductivity was observed for all samples.

The results were consistent when it came to F samples with heightened placental content. The measured values fall within the same range. The graphs of materials without COL and those incorporating 0.03 g of COL show almost identical behaviors. The samples with 0.06 g of COL exhibited slightly lower ionic conductivity values during the entire incubation period. The highest values of the tested parameter were achieved on day 7 of incubation.

The negligible changes in the conductivity of the PBS solution observed during the test lead to the conclusion that the materials are stable in this physiological fluid. However, a slight rise in sample conductivity could indicate material degradation.

### 2.2. Determination of Sorption Capacity

**Figure 3:**
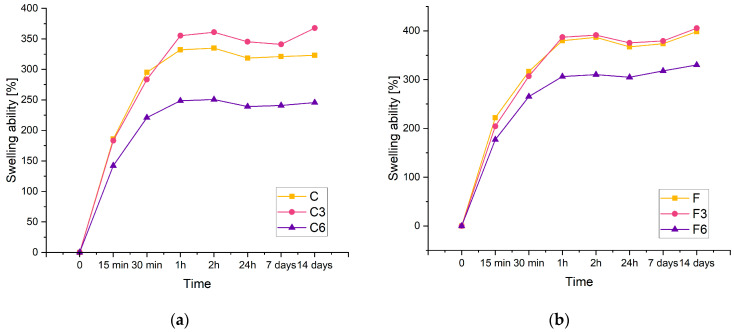
Kinetic of swelling of composites with (**a**) 0.15 g or (**b**) 0.30 g of placenta incubated in PBS solution.

The swelling rate of hydrogels is an important parameter because it controls the release pattern of solvents or active ingredients such as antibiotics. Figure 3 displays the swelling kinetics of the prepared materials containing 0.15 g of placenta (a) and 0.30 g of placenta (b). From the results, it can be concluded that the investigated composites have a significant swelling potential. Biomaterials carrying 0.15 g of placenta are identified by the maximum swelling ratio of approximately 360% and 405% for composites containing 0.3 g of placenta. Increasing the amount of placenta has been found to improve sorption properties. This study observed that the swelling capacity of each material increases as the swelling time increases. In a recent study, a similar phenomenon was reported in polyacrylamide–cellulose nanocrystal hydrogels [34].

The results showed that the inclusion of COL had an effect on the sorption capacity of the material. Despite the amount of placenta present in the composite, the samples with lower COL content have a higher swelling ability compared to matrices containing 0.06 g of COL. Additionally, samples lacking COL and those containing 0.03 g of COL exhibit analogous sorption properties. Biomaterials with the highest COL content display the poorest sorption properties for both types of samples.

The composites doubled in volume after 14 days of incubation. This suggests that the resulting materials have potential for use as absorbent dressings.

### 2.3. Degradation

Degradation analysis was performed on the basis of the weight loss of the samples after 14 days of incubation in three physiological fluids: SBF, Ringer’s fluid, and PBS. The degree of degradation helps determine the durability of the material and its usefulness for biomedical applications. The exact results are presented in Table 1. It was found that samples with less placenta, i.e., sample C, had less weight loss after incubation in each of the incubation fluids compared to composites containing more placenta. It was also noted that the presence of COL as well as increases in the amount of this component had an effect on reducing the weight loss of the biomaterials. In general, the lower the mass loss of the material, the greater the chances of obtaining a better durability of these samples. The most satisfactory results for the samples were obtained in SBF solution.

### 2.4. Fourier Transform Infrared Spectroscopy Analysis

After analyzing the measurements, composite matrices C6 and F6 were taken up for further testing because they displayed the most beneficial parameters. FT-IR spectrophotometric analysis was used to determine the chemical composition of the resulting biomaterials.

**Figure 4:**
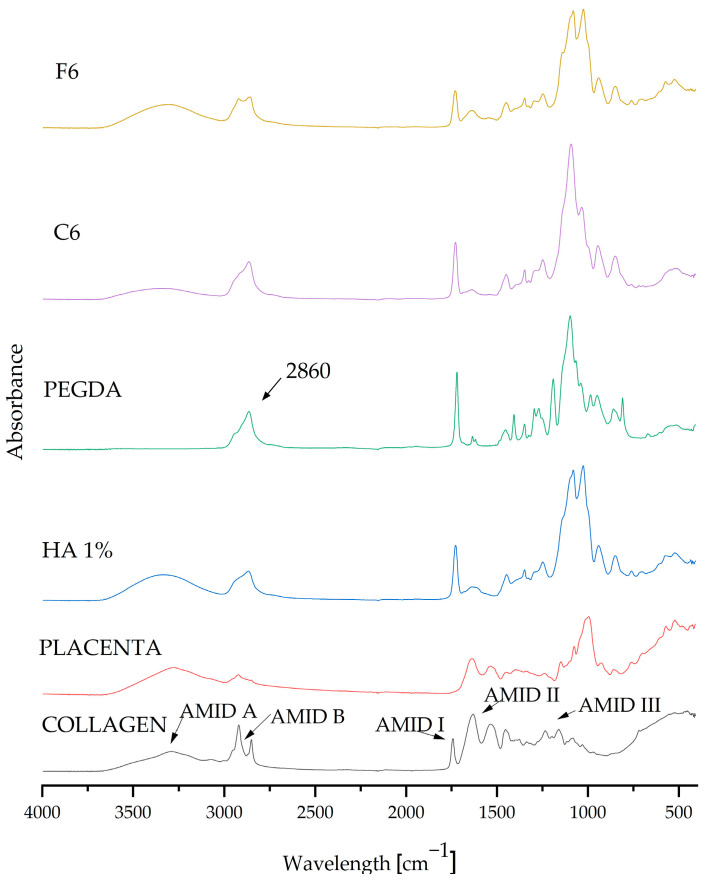
FT-IR spectra of pure ingredients and composites C6 and F6.

FT-IR examination was carried out as a part of the qualitative assessment and confirmation of the presence of pure components in the resulting coatings. The graph in Figure 4 presents the absorption spectra of both the pure components and the composite materials C6 and F6. The spectra of all samples (coating compositions, which were selected as carrying the highest potential) are very similar, and their spectra match the characteristic peaks of the substances in question. The composition of the composite coatings showed that all of the components present were pure. The FT-IR spectra display the characteristic vibrations (stretching and bending) of functional groups. The visible absorption band at 2860 cm^−1^ belongs to the crosslinking agent PEGDA 700, representing C–H stretching vibrations [5]. Characteristic peaks originating from COL were also visible and were classified as amide peaks; we observe a peak for amide I at 1740 cm^−1^ and a peak for amide II at 1640 cm^−1^, while two weaker vibrations for amide III are centered at 1230 cm^−1^ [35]. The characteristic spectrum at 1108 cm^−1^ can be attributed to C–N stretching vibrations belonging to aliphatic amides originating from the placenta [36].

### 2.5. Morphology Analysis

The biomaterials were incubated in physiological fluid at 36.6 °C for 14 days, which corresponds to human body temperature. After the incubation period, a microscopic analysis was conducted to assess the composite surface for any abnormalities. The surface of the composites was analyzed before and after the incubation period to determine the changes that occurred on the surface due to the interaction between the sample and the incubation fluid. These observations can help determine the bioactivity of the material.

**Figure 5:**
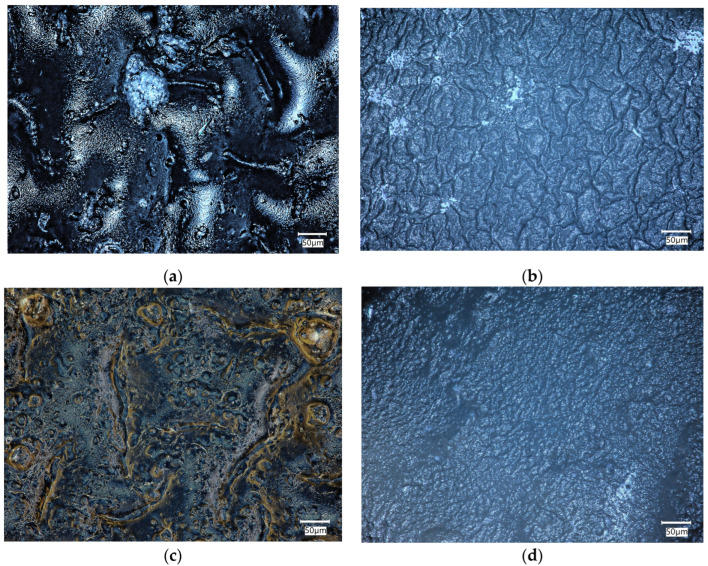
Microscopic images of C6 (**a**,**b**) and F6 (**c**,**d**) samples before (**a**,**c**) and after (**b**,**d**) incubation in SBF solution.

Figure 5 compares the surfaces of biomaterials C6 and F6 before and after incubation in SBF solution.

**Figure 6:**
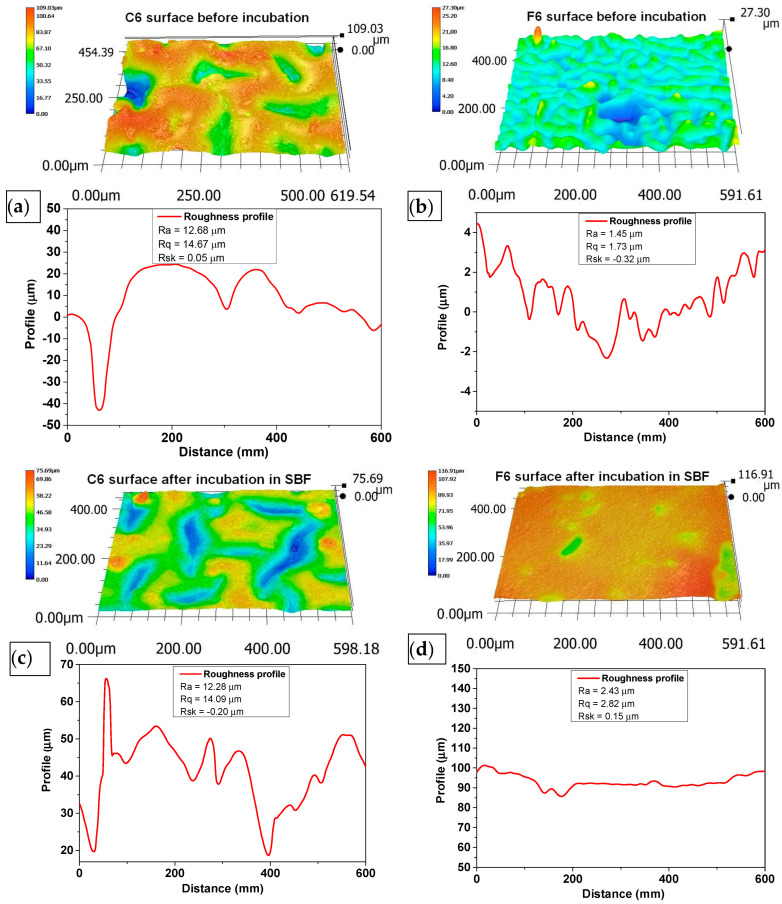
Three-dimensional reconstructions of the surface of C6 (**a**,**b**) and F6 (**c**,**d**) samples before (**a**,**c**) and after (**b**,**d**) incubation in SBF solution. Topography was obtained using reconstruction mode of optical microscope based on pictures taken with 500× magnification.

The morphology of the surface layer of the fabricated composites was visualized using a 3D reconstruction tool on an optical microscope. Figure 5 depicts images of the surface of the samples before and after incubation in SBF liquid. Three-dimensional reconstructions were made on the basis of the 2D images in Figure 6.

The surface roughness was determined according to ISO 21920-2:2021 [37] using a line distance of 60 um. The roughness profile, including parameters Ra (roughness), Rq (kurtosis), and Rsk (skewness), numerically describes the topography of the measured surfaces. Ra characterizes the departures of the roughness profile from the mean line, and Rq is the rms (root mean square) parameter corresponding to Ra. The skewness (Rsk), which describes the asymmetry of the profile about the mean line, shows a tendency to have positive or negative values. The mean value and deviation were determined from at least three repetitions of measurement at different spots on the sample.

An analysis of the surface of the samples with 0.06 g of COL after incubation in SBF did not reveal any unfavorable structures, cavities, or other defects. The absence of such defects qualifies the biomaterials as potential dressings. Fragments of COL and placenta are observed in the surface images of both C6 and F6 materials; however, no major agglomerations of these components were observed. The surfaces of samples C6 and F6 are rough and wavy, with a similar roughness ranging from 12.28 to 12.68 µm and a similar root mean square ranging from 14.09 to 14.67 µm. Only skewness is dissimilar in describing the asymmetry, which is positive in sample C6, consisting predominately of peak asperities visible in Figure 6a. The skewness of sample F6 is negative, representing surfaces that consist primarily of valleys, which can be clearly observed in Figure 6c.

The surfaces of both samples after incubation become smoother and less wavy; the roughness parameter is reduced by one order of magnitude, which is followed by Rq. The skewness of sample C6 after incubation is negative, which is consistent with valleys, while for sample F6, the skewness is positive. It can be concluded that the smoothening of the surface may be related to the nature of the physiological fluid in which the incubation took place. It should be emphasized that this structure is not related to material loss, since degradation analysis indicates minimal degradation in this physiological fluid.

### 2.6. Biological Evaluation of Cells Exposed to Biomaterials

The materials with the most optimal physicochemical properties (C6, F6, HA (hyaluronic acid), HA + COL (hyaluronic acid and collagen)) were selected for cellular in vitro studies. 

#### 2.6.1. Viability and Morphology of L929 Fibroblasts

**Figure 7:**
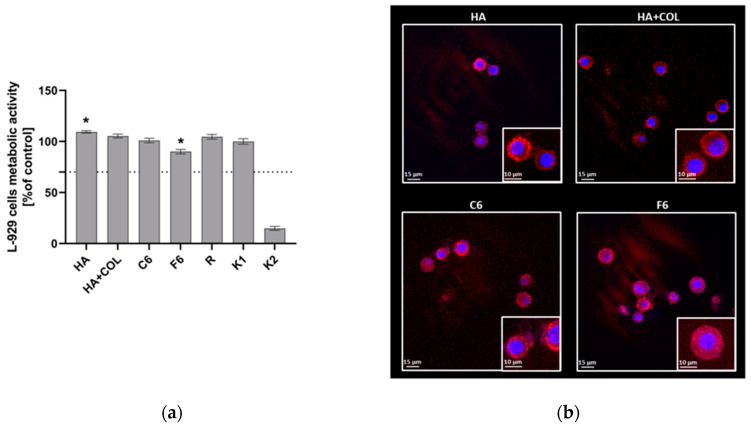
(**a**) The metabolic activity of L929 fibroblasts was assessed using MTT reduction assay after 24 h of incubation with biomaterials. Data are presented as mean viability + SD compared to control (cells in medium); assays were performed in triplicate. L929: K1 is the viability control (cells in culture medium without the test sample) and K2 is the cytotoxicity control (cells were treated with 2% saponin). The commercially available biomaterial, which consisted of tubing samples from the blood collection set, was used as a reference (R). The dotted line represents the ISO criterion for cell viability (70%). * *p* < 0.05 indicates statistically significant differences between biomaterials, and K1 was based on the one-way ANOVA (Dunnett’s) evaluation results. (**b**) The colonization of composites (HA, HA + COL, C6, and F6) by L929 cells after 24 h incubation. Cells were stained with Texas Red-phalloidin (red, F-actin) and 40,6-diamidino-2-phenylindole (DAPI) (blue, nuclei). Leica Application Suite X (LAS X; Leica Microsystems) was used for cell imaging. Each panel represents 2D pictures of biomaterials.

To assess the cytocompatibility of HA, HA + COL, C6, and F6, the MTT reduction assay was performed using L929 fibroblasts in the milieu of fabricated biomaterials. As shown in Figure 7, the viability of murine L929 fibroblasts exposed to HA, HA + COL, C6, and F6 biomaterials reached 109.34 ± 1.18%, 105.27 ± 1.9%, 100.9 ± 2.5%, and 90.02 ± 2.2%, respectively. Thus, all tested composites do not negatively affect cell activity. However, the HA composite significantly enhanced the metabolic activity of L929 cells compared to control cells (K1, 99.98 ± 2.6, *p* = 0.01). On the contrary, we observed a statistically significant reduction in the metabolic activity of fibroblasts cultured with the F6 biomaterial (*p* = 0.01) compared to K1. 

The adhesion of L929 fibroblasts and their expansion on the surface of composites after 24 h in culture was also investigated. The composites were effectively colonized by fibroblasts, as demonstrated in Figure 7. We noticed no differences in the number or condition of cells that colonized the modified biomaterials (C6 and F6) and control composites (HA and HA + COL). Moreover, morphological alterations were not observed either.

#### 2.6.2. Interaction with THP1-Blue NF-κB Human Monocytes

**Figure 8:**
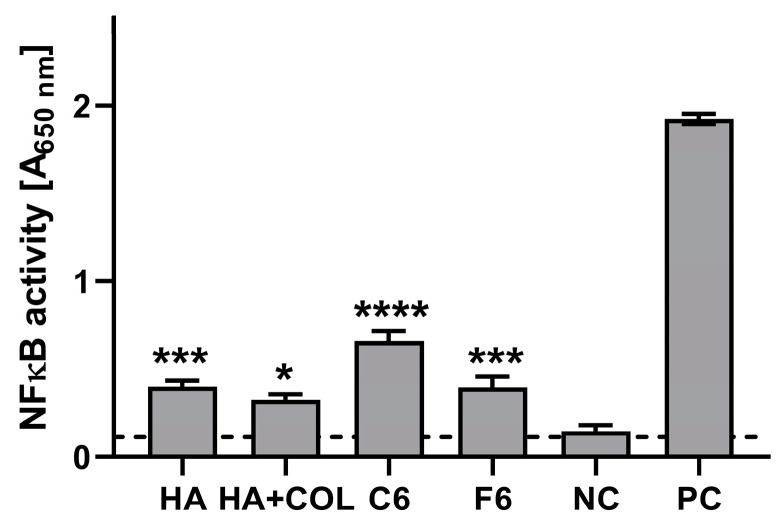
The NF-κB induction measured in THP1-Blue™ NF-κB monocytes exposed to biomaterials for 24 h. The negative control (NC) of the monocyte’s activation (cut-off line) consisted of cells incubated without composites. The positive control (PC) of the monocyte’s activation consisted of monocytes stimulated with LPS from *E. coli* (100 ng/mL). Data are presented as mean and SD of assays performed in triplicate. The differences (in comparison to NC) that are considered significant (*p* < 0.05) are marked with * (* *p* = 0.01, *** *p* = 0.002, **** *p* < 0.001), based on the one-way ANOVA (Dunnett’s) results.

The stimulatory effects of the composites (HA, HA + COL, C6, and F6) were determined via activation of the NF-κB pathway in THP1-Blue monocytes. As shown in Figure 8, after a 24 h incubation of monocytes in the milieu of biomaterials, stimulation above the cut-off value (determined for NC) was achieved for all tested composites. Compared to the untreated cells (0.114 ± 0.057), HA, HA + COL, C6, and F6 significantly induced the activation of monocytes (0.401 ± 0.139, *p* = 0.002; 0.326 ± 0.124, *p* = 0.01; 0.661 ± 0.228, *p* < 0.0001; and 0.398 ± 0.239, *p* = 0.002, respectively).

## 3. Discussion

We conducted a 14-day in vitro study to determine how the composites interacted with the solutions used for incubation. The presented studies confirm the potential use of the fabricated biomaterials as dressings. The study also confirmed that the addition of components, i.e., COL and placenta, promotes cytocompatibility, immune cell stimulation, and cell adhesion to the biomaterial.

The prepared composites reacted with the incubation liquid, which was confirmed by the change of the values of such parameters as pH and electrolytic conductivity. The pH and conductivity analyses indicate the stable behavior of the samples in PBS. All the tested samples showed similar degradation rates and only a slight change in pH during the incubation process, indicating their stability. The study of conductivity shows a slight increase in the values of this parameter during the initial phase of the incubation. However, these differences are so insignificant that they do not affect the stability of the materials that were produced.

The tests of the sorption properties of the composites clearly show that the addition of COL affects their swelling capacity. In fact, the addition of COL reduces the sorption capacity of the samples compared to those without this component. In addition, samples containing 0.06 g of COL have a significantly lower swelling ratio than those containing 0.03 g of COL. These relationships are probably due to the type of COL used to synthesize the materials in question. We decided to use solid COL, which fills the spaces between the polymer chains, resulting in reduced fluid sorption. Fibrous COL, a natural polymer that is insoluble in water, was used in the present study. It is soluble in acetic acid, but the addition of COL in acid could affect the biocompatibility of the synthesized composites. The swelling parameters showed favorable changes during the incubation process. They provide information about the release of active substances into the body, which in turn indicates the possibility of using the material as a carrier of active substances. The highest swelling coefficients were obtained by composites that did not contain COL as well as composites that contained 0.03 g of this component. The weight of the samples almost doubled after the incubation process. This result indicates the potential use of biomaterials as absorbent dressings.

The results of the in vitro biological evaluation confirmed the in vitro cytosafety of the tested biomaterials. The murine fibroblasts maintained in vitro viability of at least 70% after exposure to each composite, which means all composites met this ISO criterion (ISO 10993-5:2009 [38]). No significant differences were observed in the number or condition of the cells after exposure to the different biomaterials. Furthermore, morphological alterations were not observed, indicating that the surface properties of the composites support cell adhesion and growth. L929 fibroblasts can effectively adhere to and even proliferate on the composite surfaces, which is critical in the early stages of the wound healing process [39].

The healing process can be divided into four main phases: coagulation and hemostasis, inflammation, proliferation, and wound remodeling with scar tissue formation [40]. It was shown that high molecular weight HA displays anti-inflammatory and immunosuppressive properties. In contrast, low molecular weight HA is a potent pro-inflammatory molecule [41]. Therefore, we have studied whether HA, placenta, or COL biomaterials cause the activation of monocytes, which are innate immune cells that are recruited to the site of tissue injury and, after translocation to the injured tissue, become macrophages ready to phagocytose microorganisms and remove damaged cells. We used THP1-Blue™ cells, which, upon activation of the NF-κB transcription factor, produced secreted embryonic alkaline phosphatase (SEAP), which is an indicator of monocyte activation. We have shown that the tested composites activate monocytes, which may indicate their beneficial properties in the first phases of wound healing. Because of these properties, it is possible to reduce the risk of contamination with microorganisms and enable the effective initiation of the early inflammatory phase, which is crucial for wound healing.

Further studies are needed to explore the underlying mechanisms of these observed effects and assess the long-term activity of monocytes and fibroblasts when they come into contact with these biomaterials.

## 4. Materials and Methods

### 4.1. Reagents

Polymer matrices were prepared using bovine collagen (COL) from Sigma-Aldrich (CAS: 9067-32-70); hyaluronic acid (HA) (CAS: 9007-34-5); sheep placenta; 2-hydroxy-2-methylpropionate, used as a photoinitiator from Sigma-Aldrich; and poly(ethylene glycol) (PEGDA) Mn 700, used as a crosslinking agent from Sigma-Aldrich (Darmstadt, Germany).

### 4.2. Preparation of Composite

**Figure 9:**
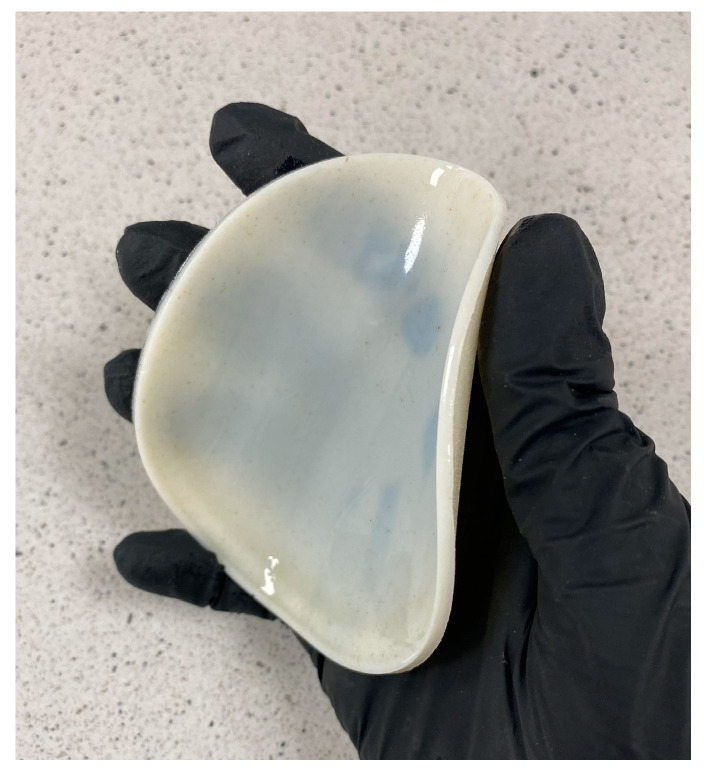
Cross-linked flexible material with a diameter of 10 cm and a thickness of 3 mm.

The first step was to prepare a 1% HA solution, and then every 10 mL was mixed with the ingredients, which were added one by one according to the proportions shown in Table 2. After adding the crosslinking agent and photoinitiator, the ingredients were mixed thoroughly using a magnetic stirrer IKA model RCT ST (IKA-Werke, Staufen, Germany). The resulting mixture was poured into 10 cm petri dishes, which were then placed under a UV lamp for 4 min (Medilux lamp type UV 436 HF, Medilux, Korntal-Münchingen, Germany, 220 V, 60 Hz) to allow the photocrosslinking process to take place. The matrices were left to dry [16]. This technology enables the production of flexible materials. An example of the crosslinked composite is presented in Figure 9.

### 4.3. Electrochemical Analysis

The experiment was carried out using completely cross-linked, disc-shaped samples of mass 1 g. They were placed in sterile containers containing 100 mL of the following incubation fluid: PBS (phosphate-buffered saline). The resulting composite materials were incubated in vitro in a POL-EKO incubator (model ST 5B SMART, Wodzisław Śląski, Poland) at 36.6 °C for 14 days. To evaluate the bioactivity of the composites, changes in pH values of the fluid were monitored. The measurements were taken at room temperature (24 °C) on a laboratory bench. Potentiometric measurements were carried out at 1, 3, 7, 10, and 14 days of incubation. Additionally, conductometric analysis was performed to evaluate the interaction between the samples and the incubation fluid. The pH and electrical conductivity values were measured using an Elmetron CX-701 multifunctional device (Zabrze, Poland).

### 4.4. Determination of Sorption Capacity

The composites were subjected to a sorption study to determine the ability of the resulting material to absorb various substances [16]. It is possible to evaluate the material’s swelling using this method. The experiment involved 1 g samples, which were placed in sterile containers, and then each sample was immersed in 100 mL of physiological fluid. The samples were incubated at 36.6 °C for 14 days. After the specified incubation time, the materials were taken out, drained of excess liquid, and then weighed. The swelling factor was calculated based on the following formula:

(1) $$Swelling\;ability=\frac{m_{1}-m_{0}}{m_{0}}\;\cdot \;100\%$$

where *m*_0_ is the dry sample mass and *m*_1_ is the mass of the specimen at a specified time of incubation.

### 4.5. Degradation

The level of degradation was estimated based on the loss of the initial mass of the sample after a 14-day incubation in the physiological fluids.

### 4.6. Fourier Transform Infrared Spectroscopy Analysis

The FT-IR (Fourier transform infrared spectroscopy) method enables the identification of substances and the analysis of complex mixtures without the need to separate them first by detecting characteristic functional groups. Spectrometers detect infrared radiation absorbed by molecules, which excites electrons [42,43]. In this study, measurements of the infrared spectrum were carried out in the wavelength range from 400 to 4000 cm^−1^ (32 scans at 4.0 cm^−1^ resolution) for pure samples, such as HA, placenta, COL, and cross-linking agent, as well as composites; for this purpose, a Thermo Scientific Nicolet iS5 spectrophotometer was used (Thermo Scientific, Loughborough, UK) with an iD7 ATR accessory.

### 4.7. Morphology Analysis

Optical microscopy enables precise assessments of composite surface quality, including structural analysis and defect detection. A VHX Series Digital Microscope (Keyence, Osaka, Japan) was used to obtain images of surface morphologies. It was possible to achieve a total image resolution of 4000 pixels (H) × 3000 pixels (V) in 4 K mode using the high-performance camera that was provided. Observations at magnifications from 20× to 2500× were possible with the high-resolution HDR function. Additionally, depth composition of low-contrasting parts or parts with significant height variations was performed. The multi-lighting function was used to detect the morphology of the coatings presented in Figure 3. Measurements of the roughness profile were taken using a 4K CMOS sensor of VHZ-7000 series (Keyence, Osaka, Japan), which performed the 2D and 3D measurements. Tests were conducted on both samples pre- and post-incubation for 14 days in physiological fluid.

### 4.8. Cell Culture Conditions

The cytocompatibility of the resulting biomaterials was evaluated according to the ISO 10993-5:2009 [38] guidelines. The L929 mouse skin fibroblasts were obtained from the American Type Culture Collection (ATCC, Manassas, VA, USA). Before the experiments, fibroblasts were cultivated in Roswell Park Memorial Institute (RPMI)-1640 medium supplemented with 10% heat-inactivated fetal bovine serum (FBS; HyClone Cytiva, Marlborough, MA, USA), penicillin (100 U/mL), and streptomycin (100 μg/mL) (Sigma-Aldrich, Darmstadt, Germany) in a humidified 5% CO_2_ atmosphere at 37 °C within the cell culture incubator. Confluent cell monolayers were detached from the culture vessel with 0.05% trypsin–EDTA solution (Gibco, Waltham, MA, USA) for subculturing and suspended in the culture medium. Cell viability and density were determined using a Burker chamber (Blaubrand, Wertheim, Germany) and the trypan blue exclusion assay. Cells were included in the experiments only if their viability exceeded 95%.

### 4.9. Cell Viability Assay

To determine the viability of L929 fibroblasts after 24 h of incubation with each biomaterial (HA, HA + COL, C6, F6), a 3-(4,5-dimethylthiazol-2-yl)-2,5-diphenyltetrazolium bromide (MTT) reduction assay was performed as described in the previous paper [44]. Shortly, after overnight incubation of the cells with the tested biomaterials, 20 μL of MTT (5 mg/mL) was added to each well. After 4 h incubation (5% CO_2_, 37 °C, >90% humidity) supernatants were replaced with 100 μL of DMSO (dimethyl sulfoxide). After a brief incubation at room temperature with constant delicate shaking, the absorbance was measured at 570 nm using a Multiskan EX reader (Thermo Scientific, Waltham, MA, USA).

### 4.10. Visualization of Cell Morphology and Expansion on Composites

To visualize the ability of the fibroblasts to adhere to and expand on the surface of the tested biomaterials, L929 cells were seeded on the composites as described previously [45]. Briefly, cells (5 × 10^5^ L929 cells) were placed on every composite and incubated for 24 h in a humidified 5% CO_2_ atmosphere at 37 °C. Next, biomaterials were washed with phosphate-buffered saline (PBS) and fixed with 3.7% paraformaldehyde (Sigma-Aldrich, Saint Louis, MO, USA) for 20 min at room temperature. The nuclei were stained with 300 nM 2-(4-amidinophenyl)-1H-indole-6-carboxamidine (DAPI) and the actin filaments with phalloidin conjugated with iFluor 594 (Cayman Chemical, Ann Arbor, MI, USA). The confocal laser scanning microscopy platform TCS LSI (Leica Microsystems, Frankfurt, Germany) was used for microscopic imaging. Samples were imaged with the following wavelength values of excitation and emission: 405 and 430–480 nm for DAPI and 590 and 615–630 nm for iFluor 594 conjugated antibody. Leica Application Suite X (LAS X; Leica Microsystems) was used for cell imaging. Confocal analysis was performed in the Laboratory of Microscopic Imaging and Specialized Biological Techniques at the Faculty of Biology and Environmental Protection at the University of Lodz, Poland.

### 4.11. NF-κB Activation

The THP1-Blue™ NF-κB human monocytes (containing the NF-κB-inducible SEAP reporter construct) obtained from InvivoGen (San Diego, CA, USA) were used to assess the activation of the NF-κB signal transduction pathway in the tested samples, as described previously [46]. Briefly, human monocytes were cultured in RPMI medium supplemented with 10% heat-inactivated FBS, 25 mM 4-(2-hydroxyethyl)-1-piperazineethanesulfonic acid (HEPES), 100 U/mL penicillin, 100 μg/mL streptomycin, 2 mM glutamine, and selective agents (100 μg/mL normocin and 10 μg/mL blasticidin) in a humidified 5% CO_2_ atmosphere at 37 °C. A total of 200 µL of cell suspension (1 × 10^6^ cells/mL) was added to a 96-well cell culture plate and stimulated with all tested biomaterials for 24 h (5% CO_2_, 37 °C, >90% humidity). Next, SEAP production was quantified after combining 20 μL of cell-free supernatant with 180 μL of QUANTI-Blue™ buffer (InvivoGen, San Diego, CA, USA) and incubating the mixture at 37 °C with 5% CO_2_ for 4 h. The absorbance was measured at 650 nm using a Multiskan EX reader (Thermo Scientific, Waltham, MA, USA). 

## 5. Conclusions

The present study describes the synthesis of dressings that can potentially promote epidermal regeneration. The developed technique was based on the use of UV light and enabled us to obtain hydrogel composite coatings under UV light. The selected parameters, such as crosslinking time and types of crosslinking agents and photoinitiators, made it possible to obtain finished materials with satisfactory organoleptic properties (without roughness or holes, fully continuous, and crosslinked). Physicochemical analysis, incubation studies, and cytotoxicity testing of the proposed solution were performed. Films based on HA and COL with placental material showed significant swelling, moisture absorption, and mechanical properties, which are ideally required for a good wound dressing preparation. The changes in swelling parameters observed during the incubation process suggest that the material could be used as a carrier for active substances. In vitro studies indicate that biomaterials interact appropriately with artificial incubation fluids, and the addition of COL and placenta increases their biocompatibility and cell adhesion. The in vitro biological evaluation confirmed the cytological safety of the tested biomaterials. L929 fibroblasts adhered and proliferated effectively on the surfaces of the composites, which is crucial in the early stages of the wound-healing process. Furthermore, the study has demonstrated that biomaterials that contain placenta and collagen compositions have a significant contribution towards the activation of monocytes. Overall, the prepared hydrogels with placenta additives represent a new idea that could be a promising medical material for accelerating wound healing. Therefore, the obtained results, considering the potential of the presented biomaterials, suggest the necessity of further research, especially in vivo.

## Figures and Tables

**Table 1:** Weight loss [g] of samples after 14 days of incubation.

Medium	SBF [g]	Ringer Solution [g]	PBS [g]
C	0.0188	0.0152	0.0173
F	0.0204	0.0226	0.0198
C3	0.0135	0.0151	0.0330
F3	0.0211	0.0192	0.0206
C6	0.0112	0.0124	0.0139
F6	0.0182	0.0239	0.0203

**Table 2:** Sample composition.

Sample Symbol	Placenta [g]	COL [g]	PEGDA 700 [mL]	HA 1% [mL]
C	0.15	-	1.8	10
C3	0.15	0.03		
C6	0.15	0.06		
F	0.3	-		
F3	0.3	0.03		
F6	0.3	0.06		

## Data Availability

The data that support the findings of this study are contained within the article.
